# Consideration of the Role of Vasomotion-Induced Flowmotion on Microvascular Blood Flow

**DOI:** 10.7759/cureus.89919

**Published:** 2025-08-12

**Authors:** Harvey N Mayrovitz

**Affiliations:** 1 Medical Education, Nova Southeastern University Dr. Kiran C. Patel College of Allopathic Medicine, Davie, USA

**Keywords:** arterioles, blood flow, blood velocity, flowmotion, mathematical model, microcirculation, microvasculature, skin, vasomotion

## Abstract

Background: The term "flowmotion" describes the temporally dynamic changes in microvascular blood flow or blood velocity accompanying spontaneous time-varying changes in arteriole diameters, a process termed "vasomotion." Flowmotion is readily measurable in human skin using laser Doppler flowmetry and has been studied empirically to characterize its spectral features and disease-related changes in amplitude and frequency. However, there is a lack of clarity regarding the effect on blood flow within arterioles that exhibit these time-varying diameter changes. Thus, the goals of the present study were to (1) investigate the effects of vasomotion on blood flow within arterioles using simulated sinusoidal and trapezoidal vasomotion patterns and (2) determine the difference in blood flow effects between these two vasomotion patterns.

Methods: The sinusoidal diameter was expressed as $D(t) = Do [1 + (y/2)sin (2&pi;t/T)$ where $y$ is the peak-to-peak vasomotion amplitude and $T$ is the period of oscillation. For the trapezoidal case, the diameter was expressed in terms of the Fourier series for a periodic trapezoidal waveform. The impacts of vasomotion as a function of the amplitude a were determined analytically for the sinusoidal case and numerically for both cases.

Results: Analysis indicates that sinusoidal and trapezoidal vasomotion are associated with an average blood flow that is greater than would be present in a vessel with a fixed diameter, and the trapezoidal pattern yields a greater increase than the sinusoidal pattern.

Conclusion: Analytic and numerical evaluations of sinusoidal and trapezoidal diameter variations revealed that the resultant flowmotion was associated with greater average blood flow, with the trapezoidal pattern being slightly more effective than the sinusoidal pattern. Although the impact of these dynamics on the functional aspects of the overall microvascular network, including microvascular exchange processes, was not considered in the present report, these aspects represent areas well warranted for future research.

## Introduction

The term "flowmotion" describes the changes in blood flow or velocity that accompany spontaneous changes in microvessel diameters, termed "vasomotion' [1]. There is extensive literature on measurements of flowmotion features in a wide range of experimental animals [2-7], and in humans [8-10]. In humans, a primary target for these measurements is the skin in both health and disease states [11-17], The measurement method most often used is laser Doppler flowmetry (LDF) [18-22]. By applying techniques such as wavelet [23-25] or Fourier [26-28] analysis to the acquired LDF data time series, the observed time-domain variations in skin blood perfusion have been assessed in terms of their frequency spectral content. As a result of these analyses, multiple frequency bands have been identified, each attributed to different physiological processes.

Changes in the temporal features of the flowmotion and spectral power within and among these frequency bands have been investigated in various pathological conditions [11,12,15,29-32]. However, the hemodynamic significance of changes in the flowmotion amplitude has not been thoroughly analyzed, although several detailed theoretical models have been employed to determine the extent to which the presence of vasomotion and its associated flowmotion affect actual blood flow, either positively or negatively. Farina et al. [33] used the maximum diameter of an arteriole exhibiting vasomotion as a reference and determined the effect of sinusoidal vasomotion on the hydraulic resistance. An outcome of their model indicated that the presence of the vasomotion increased the hydraulic resistance relative to that which would be present if the arteriole were maintained at its maximum diameter [33]. This finding is expected since the comparison was made to the arteriole’s maximum diameter.

Others have suggested that an active vessel undergoing sinusoidal rhythmicity will always have a lower vascular resistance than a passive one of the same average diameter [34,35]. However, microscopic observations of the vasomotion pattern that generates the flowmotion indicate that non-sinusoidal patterns are also present [36]. Such patterns may consist of diameter changes that are more trapezoidal than sinusoidal.

This study aimed to investigate the extent to which vasomotion affects blood flow within arterioles using simulated sinusoidal and trapezoidal vasomotion patterns and to determine the difference between these two patterns in terms of average blood flow.

## Materials and methods

**Figure 1:**
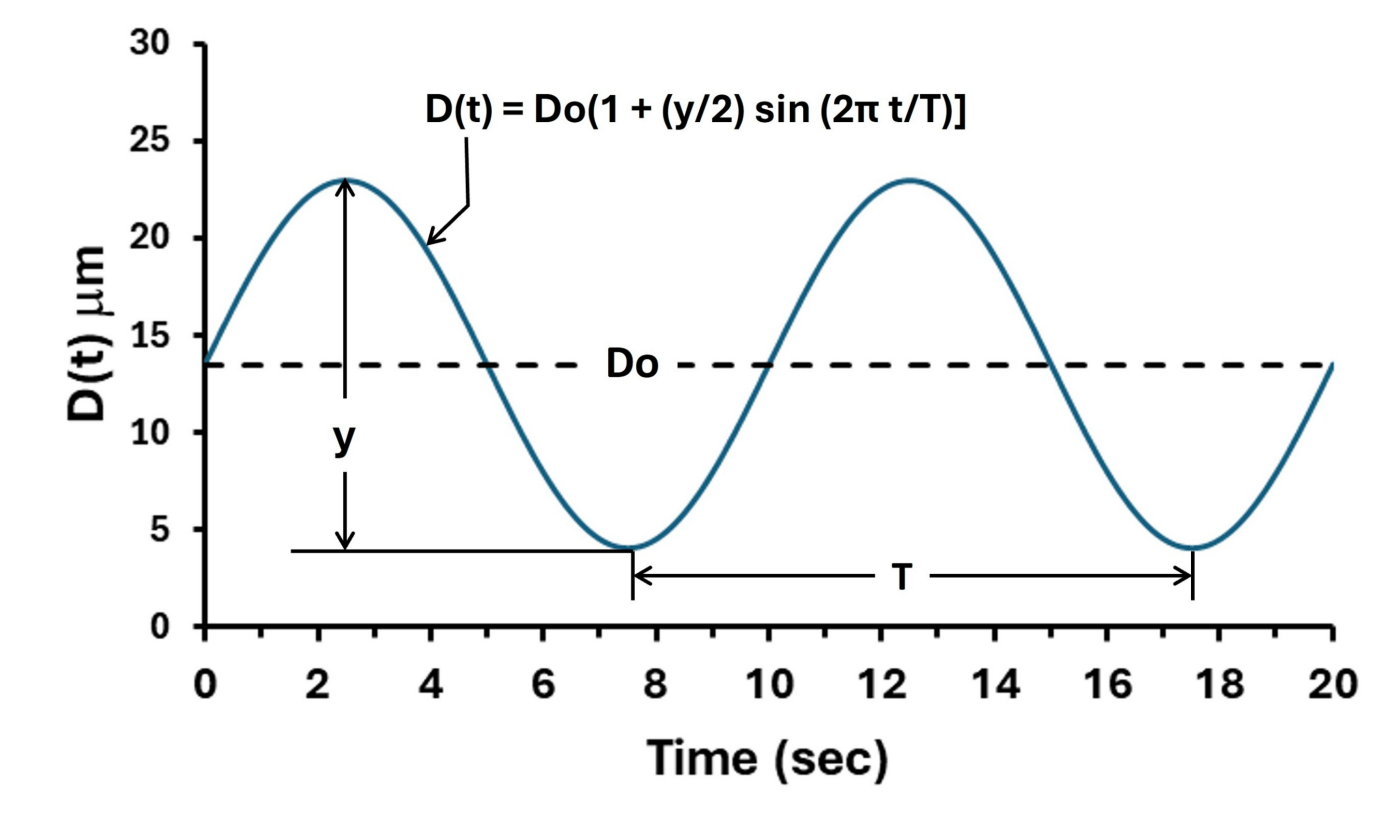
Parameters of sinusoidal vasomotion. In the figure, (y) is the peak-to-peak vasomotion amplitude, T is the period of the oscillation, and Do, the average diameter, is 13.5 μm. In this example, the period is 10 seconds, corresponding to six cycles per minute or a frequency of 0.1 Hz. The value for (y) is 1.6. The vertical scaling for D(t) approximates commonly measured arteriole diameters. A (y) value of 2.0 indicates the maximum possible vasomotion amplitude that results in blood flow stoppage during the maximum constrictive phase.

It is assumed that a blood vessel, such as a small arteriole, has a diameter $(D)$ that varies temporally around its constant mean diameter $(Do)$ as shown in Figure 1. The diameter variation is given as $D(t) = Do [1 + (y/2) sin (2&pi;t/T)$ where $y$ is the peak-to-peak amplitude and $T$ is the period of oscillation.

For laminar blood flow $(Q)$, as is the case in small arterioles, $Q$ is approximately governed by Poiseuille’s equation, which can be expressed as $Q = P&rsquo; (&pi;D^4)/128vL$. In this equation, $v$ is the blood’s viscosity, $L$ is the vessel length, and $P&rsquo;$ is the arteriole’s perfusion pressure, which, for a vessel within an extensive interconnected microvascular network, is approximately constant. The arteriole blood flow as a function of time is $Q(t) = K [D(t)]^4$, in which $K$ is $P&rsquo; (&pi;D^4)/128vL$, and the average blood flow $Q(t)avg$, averaged over a time interval $T$, is $K (1/T) &int;[D(t)]^4dt$ with limits of integration from 0 to T. If $Qo$ is the flow in a vessel that has a fixed diameter, $Do$, its average flow is $Qo = K[Do]^4$. The ratio of the average blood flow in a vessel experiencing vasomotion to the blood flow in the vessel if the diameter were constant is $&int;[D(t)]^4dt / T[Do]^4$.

The pattern shown in Figure 1 considers an arteriole that has a sinusoidal diameter variation $D(t) = Do [1 + (y/2) sin (2&pi; t/T)]$, where $y$ is the peak-to-peak amplitude of the vasomotion and $T$ is the average period of the vasomotion. Vasomotion frequencies have been reported to range from approximately 3 to 10 cycles per minute (cpm), with average periods ranging from 6 to 20 seconds. The example in Figure 1 is for an arteriole with an average diameter of 13.5 μm, consistent with skin arteriole values [37-39] displaying vasomotion at a frequency of 0.1 Hz. A trapezoidal vasomotion pattern was generated by expressing $D(t)$ via the Fourier series for a periodic trapezoidal waveform as follows:

$D(t)_{\text{trap}} = K [ \sin(t) + \sin(3t)/9 - \sin(5t)/25 - \sin(7t)/49 + \sin(9t)/81 + \sin(11t)/121 - \cdots ]$

**Figure 2:**
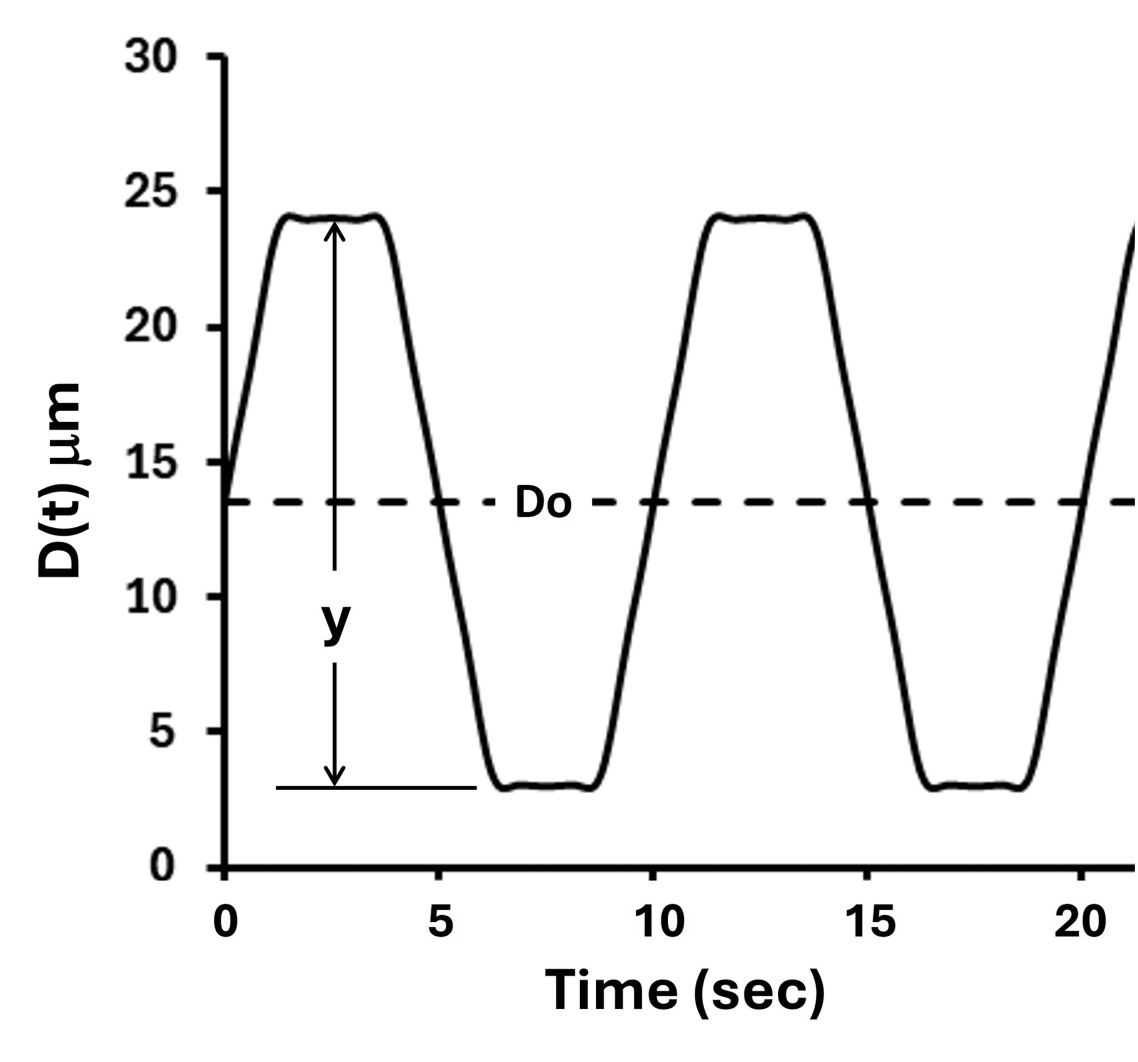
Parameters of trapezoidal vasomotion The peak-to-peak vasomotion amplitude is denoted as (y). In this example, Do is the average diameter (13.5 um), the period is 10 seconds, and the value for (y) is 1.6. A (y) value of 2.0 indicates the maximum possible vasomotion amplitude that results in blood flow stoppage during the maximum constrictive phase.

This was then scaled to the same maximum diameter as for the sinusoidal case, as shown in Figure 2.

Vasomotion effects on blood flow

The effects of vasomotion on blood flow were analyzed for the sinusoidal diameter pattern shown in Figure 1 using both analytical and numerical approaches for confirmation. The numerical approach computed the equation $[D(t)]^4= Do [1 + (y/2)sin (2&pi; t/T)]^4$for small increments of $t/T$ for varying values of $y$ in Microsoft Excel and computed the cycle average numerically as a function of y. Additionally, for the sinusoidal case, an analytical solution was formulated to express the ratio of blood flow in an arteriole experiencing vasomotion $Q(t)avg$ the blood flow that occurs without vasomotion $Q(o)$ with the same mean diameter. The details and results of this analysis are given in Appendix 1. The solution is repeated here for convenience, as equation (1) below.

Eq 1: $Q(t)avg / Q(o) = 1 + (3/4)(y/Do)^2+ (3/128)(y/Do)^4$

For the trapezoidal vasomotion case $D(t)_{\text{trap}}$was handled numerically in the same way as $D(t)$ for the sinusoidal variation to determine and compare its effect on the average $Q(t)$ versus that for the sinusoidal variation.

## Results

**Figure 3:**
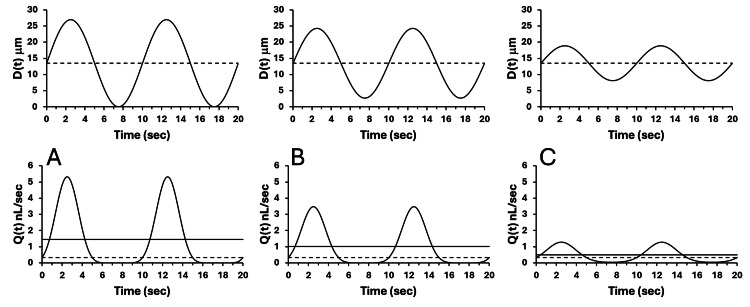
Example of vasomotion effects on flowmotion amplitude and time average flow for sinusoidal diameter variation The top panel shows diameter variations, D(t) in μm, and the bottom panel shows the corresponding flowmotion pattern, Q(t) in nL. In the top panel, the dashed line is the constant average diameter of the simulated arteriole. In the bottom panel, the solid line is the average blood flow attributable to flowmotion, and the dashed line is the average flow that would occur if the arteriole had the average diameter shown in the upper panel. In sections A, B, and C, the value of vasomotion amplitude (y) in the diameter simulation equation is 2.00, 1.60, and 0.80, respectively. As the amplitude of vasomotion decreases (A through C), so does the flowmotion, with the average flow approaching that of steady flow in the constant diameter arteriole. A (y) value of 2.0 indicates the maximum possible vasomotion amplitude that results in blood flow stoppage during the maximum constrictive phase.

Figure 3 shows the effects of varying vasomotion amplitudes on blood flow for sinusoidal diameter variations. The three vasomotion amplitudes illustrated in this figure correspond to values of $y$ = 2.00, 1.60, and 0.80, with $y$ as defined in Figure 1. The value $a$ = 2.00 corresponds to a complete closure of the arteriole during the maximum vasoconstrictive phase of the vasomotion cycle. The arteriole is scaled to have an average diameter of 13.5 μm, consistent with values reported for skin arterioles [37-39]. For illustrative purposes, the blood flow value is scaled assuming that when the arteriole's diameter is at its maximum (27 μm), it supplies blood to 100 capillaries, each of which has an average red blood cell velocity of 0.10 cm/sec and a diameter of 8 × 10^-4^ cm. Accordingly, the cross-sectional area of each capillary is 5.02 x 10^-7^ cm^2^, and each has a blood flow of 5.02 x 10^-2^ nL/sec. Thus, the maximum flow in the arteriole undergoing vasomotion would be 5.02 nL/sec as shown in Figure 3A. From this figure, it can be seen that as the amplitude of vasomotion decreases, both the maximum and average blood flow associated with the arteriole undergoing vasomotion are reduced. As the amplitude approaches zero, the flow approaches that which would be present in an arteriole with a constant diameter equal to $(Do)$.

**Figure 4:**
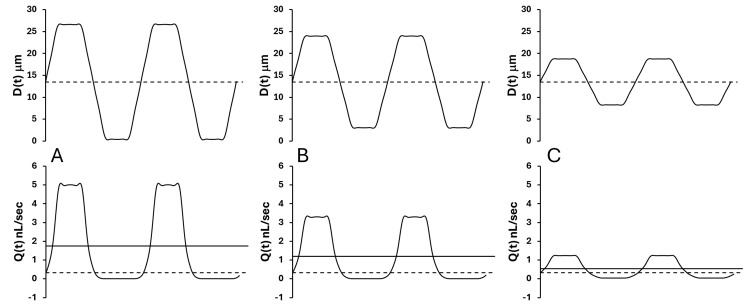
Example of vasomotion effects on flowmotion amplitude and time average flow for trapezoidal diameter variation. The top panel shows diameter variations, D(t) in μm, and the bottom panel shows the corresponding flowmotion pattern, Q(t) in nL. In the top panel, the dashed line is the constant average diameter of the simulated arteriole. In the bottom panel, the solid line is the average blood flow attributable to flowmotion, and the dashed line is the average flow that would occur if the arteriole had the average diameter shown in the upper panel. In sections A, B, and C, the value of the vasomotion amplitude (y) in the diameter simulation equation is 2.00, 1.60, and 0.80, respectively. As the amplitude of vasomotion decreases (A through C), so does the flowmotion, with the average flow approaching that of steady flow in the constant diameter arteriole. A (y) value of 2.0 indicates the maximum possible vasomotion amplitude that results in blood flow stoppage during the maximum constrictive phase.

A similar pattern occurs when the vasomotion process is characterized by trapezoidal diameter variation, a pattern that may more closely resemble the diameter variation observed [36]. The effect on the resultant blood flow is illustrated in Figure 4. The trapezoidal diameter variation is scaled to the same maximum diameter as for the sinusoidal case. From this figure, it can be observed that, as in the case of sinusoidal variation, the time-varying blood flow average approaches the steady value that occurs without vasomotion, as the vasomotion amplitude decreases. 

**Figure 5:**
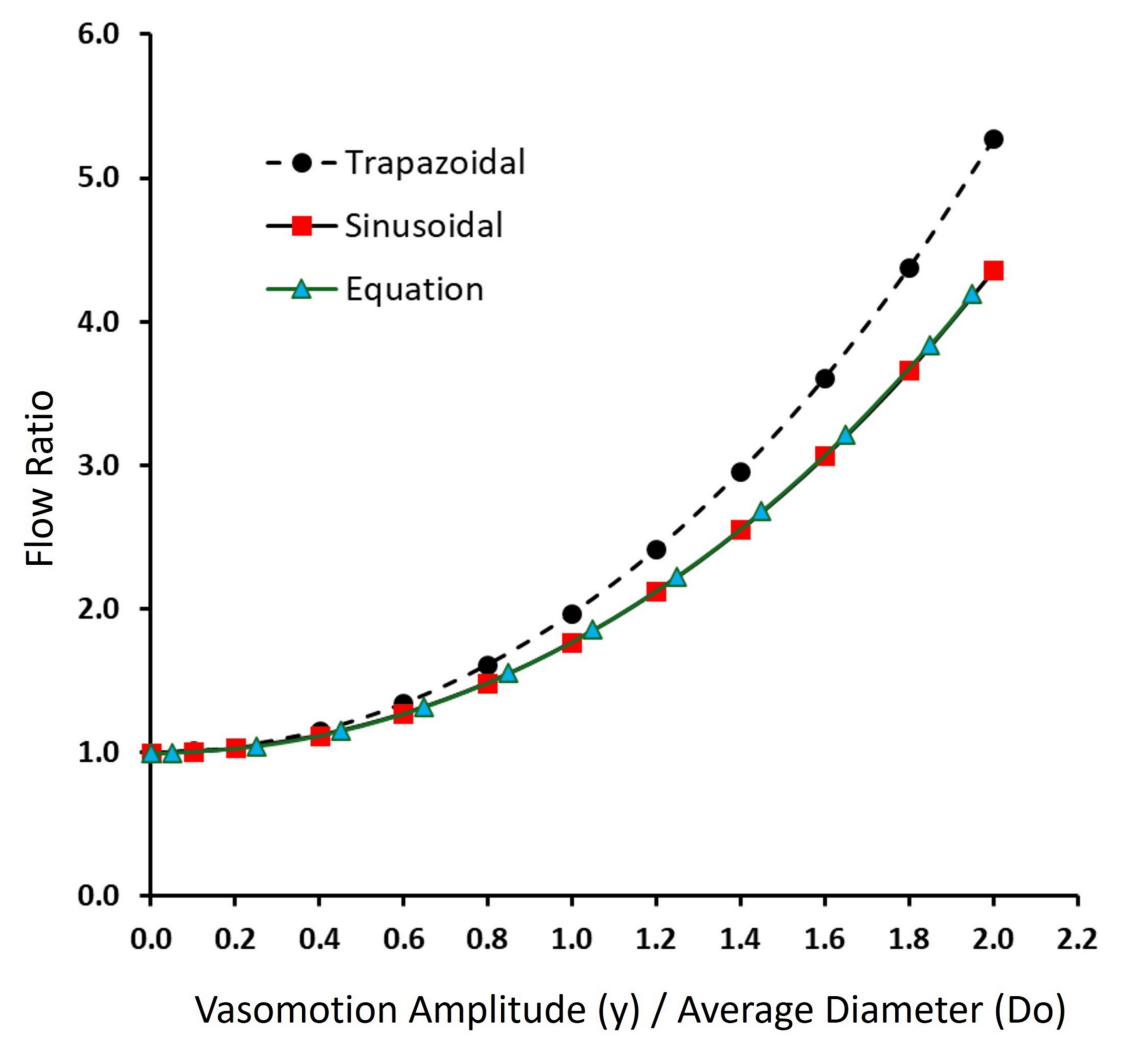
Flow ratio dependence of vasomotion amplitude The flow ratio is defined as the ratio of the average blood flow in a vessel displaying vasomotion to the blood flow in a vessel with a constant diameter equal to the same average diameter. The red squares are from the numeric simulation of the sinusoidal diameter variation, and the green triangles are from the analytic equation (A3) from Appendix 1. The black circles are numerically determined for the trapezoidal vasomotion pattern. The agreement between the analytic and numerical analysis of the sinusoidal pattern is notable. The trapezoidal variations indicate a slightly greater vasomotion effect than that for sinusoidal variations. A y/Do ratio of 0.0 indicates zero vasomotion. A (y/Do) value of 2.0 indicates the maximum possible vasomotion amplitude that results in blood flow stoppage during the maximum constrictive phase.

The magnitude of vasomotion-related effects changes with amplitude and differs slightly for the trapezoidal pattern compared to the sinusoidal, as shown in Figure 5. This figure is a graphic representation of the flow ratio $Q(t)avg/Q(o)$ as a function of the relative vasomotion amplitude $(y/Do)$. An arteriole without vasomotion would have a $y/Do$ value equal to 0 for which the flow ratio would 1.0. As the relative amplitude increases, so does the flow ratio, being slightly greater for the trapezoidal diameter variation.

## Discussion

The primary goal of the present study was to investigate the impact of microvascular vasomotion patterns on average blood flow within the arteriole, demonstrating the flowmotion. Analytic and numerical evaluations of sinusoidal and trapezoidal diameter variations revealed that the resultant flowmotion was associated with increased average blood flow, with the trapezoidal pattern being slightly more effective than the sinusoidal pattern.

The analytic approach used for the sinusoidal pattern in the present study paralleled that reported by Meyer et al. [40], which was part of their criticism of a prior formulation by Gratton et al. [41]. However, the present analysis differs in that it expresses the results in terms of peak-to-peak diameter variations, which is most easily measured experimentally and approaches the analysis from the hemodynamic conductance viewpoint rather than vascular resistance. Nonetheless, the analytical results have the same form but with appropriately different numerical coefficients as expressed by equation A3 for the normalized form and equation A4 for the absolute average flow. In addition, the numerical and analytic solutions yield the same results as demonstrated by the graphic shown in Figure 5 for the sinusoidal pattern.

An alternative approach to the present studied problem was taken by Slaaf et al. [35], who introduced the concept of an "effective" diameter and suggested that an organ receives the same amount of blood flow regardless of whether its arterioles exhibit vasomotion. They suggested that the use of the effective diameter concept eliminated ambiguity in the interpretation of vasomotion effects and cited an example to illustrate the difficulty in interpreting between two vasomotion pattern cases. The example cited considered the difference between a sinusoidal vasomotion pattern in which an arteriole had either a diameter of 25 μm with a 5 μm vasomotion amplitude (y) or had a 22 μm average diameter around which there was a vasomotion amplitude of 22 μm. Use of equation A4 of the present work indicates no ambiguity, demonstrating that the larger amplitude vasomotion case is associated with the greater average blood flow in the cited case.

Study limitations

Important considerations that were not the focus of the present work and therefore not addressed at this time were the physiological source of the vasomotion [42,43], the overall impact on network blood flow if multiple microvascular vessels exhibited either in-phase or out-of-phase vasomotion patterns [44,45], and the effect of vasomotion on other aspects of microvascular regulation, including network resistance distribution and transcapillary exchange processes [46-48]. Furthermore, the present analyses did not account for possible effects of non-symmetrical vasomotion patterns. Additionally, in the case of the trapezoidal pattern, the results are strictly applicable to patterns with equal rise and fall times of the diameter changes. However, given the closeness between the sinusoidal and trapezoidal results, this is not considered a significant factor.

## Conclusions

Analysis of sinusoidal and trapezoidal vasomotion patterns, when present within arterioles, indicates that the induced flowmotion is associated with an average blood flow within such vessels that is greater than would be present in a vessel with a fixed diameter and that the trapezoidal pattern results in a greater increase than the sinusoidal pattern. This analysis is based on certain assumptions and applies to hemodynamic events occurring within a single arteriole embedded within a microvascular network. By considering both sinusoidal and vasomotion patterns, the results may provide upper and lower bounds on the type of experimentally observed vasomotion features. The impact of such properties on the functional aspects of the overall microvascular network, which encompasses multiple vessel vasomotion interactions and microvascular exchange processes, is not considered in this report. It is suggested that entry into these investigative paths is well warranted. The present work may provide an additional basis to aid the interpretation of findings associated with directional changes in flowmotion observed in various clinical conditions.

## Appendices

Consider the diameter $D(t)= Do+(y/2)sin(z)$ with $z = 2&pi;t/T$ corresponding to the definitions of Figure 1. The arteriole’s hemodynamic conductance with vasomotion is $G(t)=K(Do+(y/2)sin(z))^4$, and the arteriole’s conductance with fixed diameter Do is $G(o) = KDo^4$. The conductance ratio, which defines the flow ratio, is given by A1.

A1. $G(t)/G(o)=[(Do + (y/2)sin(z))/Do]^4 =[(1 + gsin(z)]^4$ with $g=(y/2)/Do$.

Expansion of the A1 form $[(1 + gsin(z)]^4$ yields equation A2 for the flow ratio.

A2. $Q(t)/Q(o) = 1 + 4gsin(z) + 6g^2sin2(z) + 4g^3sin3(z) +g^4sin4(z)$.

To find the average flow ratio, A2 is integrated term by term according to $1/2&pi;&int;f((z))d(z)$ with limits of $(z)$ from 0 to 2$&pi;$. This process yields the final flow ratio result shown in equation A3.

A3. $Q(t)avg/Q(o) = 1 + (3/4)(y/Do)^2 + (3/128)(y/Do)^4$

Equation A3 represents the blood flow ratio expressed as a function of the ratio of the amplitude ($y$) to the average diameter $Do)$ for sinusoidal vasomotion. To determine the average absolute blood flow, equation A4 would apply.

A4. $Q(t)avg = KDx [1 + (3/4)(y/Dx)^2 + (3/128)(y/Dx)^4]$

 In A4, $(Dx)$ could be considered as a diameter around which the arteriole symmetrically varied in a sinusoidal pattern. 
